# Modeling the behavior of human induced pluripotent stem cells seeded on melt electrospun scaffolds

**DOI:** 10.1186/s13036-017-0080-5

**Published:** 2017-10-23

**Authors:** Meghan E. Hall, Nima Khadem Mohtaram, Stephanie M. Willerth, Roderick Edwards

**Affiliations:** 1 Department of Mathematics and Statistics, University of Victoria, Victoria, Canada; 2 Department of Mechanical Engineering, University of Victoria, Victoria, Canada; 3 Division of Medical Sciences, University of Victoria, Victoria, Canada; 4 Department of Biochemistry, University of British Columbia, Vancouver, Canada; 5 International Collaboration on Repair Discoveries, University of British Columbia, Vancouver, Canada; 6 Centre for Biomedical Research, University of Victoria, Victoria, Canada

**Keywords:** Stem cell, Ordinary differential equation, Differentiation, Proliferation, Tissue engineering

## Abstract

**Background:**

Human induced pluripotent stem cells (hiPSCs) can form any tissue found in the body, making them attractive for regenerative medicine applications. Seeding hiPSC aggregates into biomaterial scaffolds can control their differentiation into specific tissue types. Here we develop and analyze a mathematical model of hiPSC aggregate behavior when seeded on melt electrospun scaffolds with defined topography.

**Results:**

We used ordinary differential equations to model the different cellular populations (stem, progenitor, differentiated) present in our scaffolds based on experimental results and published literature. Our model successfully captures qualitative features of the cellular dynamics observed experimentally. We determined the optimal parameter sets to maximize specific cellular populations experimentally, showing that a physiologic oxygen level (∼ 5%) increases the number of neural progenitors and differentiated neurons compared to atmospheric oxygen levels (∼ 21%) and a scaffold porosity of ∼ 63% maximizes aggregate size.

**Conclusions:**

Our mathematical model determined the key factors controlling hiPSC behavior on melt electrospun scaffolds, enabling optimization of experimental parameters.

## Background

Tissue engineering combines biomaterials and cells, creating functional structures that can replace damaged regions of tissue [1, 2]. Pluripotent stem cells can differentiate into any cell type found in an organism, making them a valuable tool for tissue engineering [1–3]. In 2006, Takahashi and Yamanaka discovered that mature cells could be reprogrammed back into a pluripotent state, which introduced the option of using patient derived stem cells as a tool for engineering tissues [4]. These cells were termed induced pluripotent stem cells (iPSCs). Controlling the differentiation of human iPSCs into functional tissues remains difficult because complicated cell signaling networks regulate this process [5–9]. Both physical and chemical cues control stem cell differentiation [5–9], and the signalling mechanisms involved may be controlled, chaotic, random, or a combination of these types [10, 11].

Stem cells can produce additional stem cells or they can differentiate, which occurs when their gene expression is altered to reflect a terminal state [8]. During the differentiation process, cells go through distinct transition to a progenitor state where they are not considered stem cells or differentiated cells [9, 10]. Distinct cellular markers, such as proteins, can distinguish the state of a cell in the differentiation process [8, 12–14]. While previously differentiation was thought to be irreversible, mature cells can revert from the terminally differentiated state into the stem state in both nature and the lab [9, 15]. While mature cells rarely revert from a differentiated state, progenitors revert back into stem cells more frequently [9, 16]. Our model considers these three cell states (stem, progenitor, and differentiated).

These different cellular populations interact through various mechanisms, including release of soluble factors and binding of membrane proteins [17]. These mechanisms activate signaling pathways that vary in their speed and effective distance. Collectively these mechanisms lead to major differences in the overall effect of the signal on stem cell behavior in terms of proliferation versus differentiation. For example, the cellular responses to contact (a mechanical process) and oxygen levels (a chemical process) would likely have different mechanisms. In tissue engineering, the scaffold provides mechanical cues to the seeded stem cells, inducing differentiation through mechanical input [5, 14, 18, 19]. Stiffness affects the type of cell resulting from differentiation: cells differentiate into neural, mesenchymal, and bone with increasing scaffold stiffness [19]. Moreover, substrate stiffness modulates how neural stem cells differentiate into mature cells of the nervous system, including neurons, astrocytes, and oligodendrocytes [5]. In addition to stiffness, porosity affects stem cell differentiation and proliferation because contact induces these processes, but excessive contact inhibits aggregate growth. Scaffold topography also influences stem cell growth and differentiation [14]. For example, fiber diameter of fibrous scaffolds affect aggregate growth: suboptimal fiber sizes inhibit cellular proliferation [14]. Here we use melt electrospinning to fabricate our biomaterial scaffolds. Melt electrospinning produces highly reproducible engineered microfiber scaffolds with controllable properties, such as fiber diameter and porosity, that provide topographical cues. Our group has done extensive work designing and fabricating such scaffolds for promoting the neuronal differentiation of pluripotent stem cells. While solution electrospinning is commonly used to fabricate scaffolds for tissue engineering applications, it presents many challenges such as the use of toxic solvents and the lack of reproducibility. In either case scaffolds can degrade over time, resulting in decreased stiffness, increased porosity, and changes in topography.

**Fig. 1:**
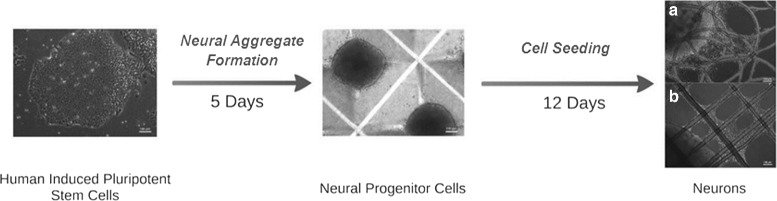
Experimental protocol for neural aggregate formation and seeding on to melt electrospun scaffolds. Human induced pluripotent stem cells (colony shown on the left) were cultured in Aggrewell plates in the presence Neural Induction Medium (center) to form aggregates of neural progenitor cells. The neural aggregates are then seeded onto melt electrospun scaffolds where they differentiate into neurons (right)

Here we model how human iPSC-derived neural aggregates grow and differentiate on biomaterial scaffolds produced by melt electrospinning poly caprolactone. The experimental procedure that precedes the aggregate seeding is outlined in Fig. 1. Our model requires the aggregates to make contact with the electrospun substrates to initiate differentiation. Previous experimental observations indicate that neural aggregates in suspension do not progress from the progenitor state to the terminally differentiated state, but seeding them upon a biomaterial substrate triggers differentiation [14].

There has been significant research into stem cell proliferation and differentiation, both experimentally and using mathematical models [2, 5–7, 10, 11, 13, 20–22]. The current work incorporates multiple intrinsic and extrinsic factors that affect stem cell population dynamics, making it distinct from previous studies. The intrinsic characteristics include cell-cell signalling, differential responses to extrinsic effects, and state-specific metabolic properties. The extrinsic properties include scaffold effects, oxygen and waste effects, depth of culture medium, and control of differentiation via growth factors. Using coarse approximations of many of these processes allows us to include more experimental properties in a single model. This model gives insight into how the experimental procedures could be altered to maximize a specific population of cells. In particular, we show that a physiologic oxygen level (∼ 5%) increases the number of neural progenitors as well as differentiated neurons compared to atmospheric oxygen levels (∼ 21%) and a scaffold porosity of ∼ 63% maximizes aggregate size.

## Methods

### Experimental methods

**Fig. 2:**
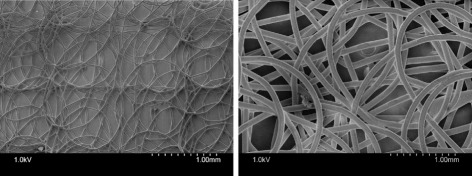
Scanning electron microscope images of loop mesh 200 (left) and loop mesh 500 (right) scaffolds

A custom-made melt electrospinning setup was used to fabricate poly-(*ε*-caprolactone) (PCL, number average molar mass (Mn) ∼ 45,000, Sigma Aldrich Chemical Co) biomaterial scaffolds [14]. Melt electrospinning was performed using nozzles with diameters of 200 *μ*m and 500 *μ*m to fabricate scaffolds referred to as loop mesh 200 and loop mesh 500, respectively [14]. Increasing nozzle size corresponds to an increase in fiber diameter and a decrease in porosity [14]. The resulting porosities were 23% for loop mesh 200 and 40% for the loop mesh 500. Figure 2 shows two examples of the final scaffolds. hiPSCs were cultured on a Vitronectin XF^TM^ matrix in the presence of TeSR^TM^-E8^TM^ medium [23], then in STEMdiff^TM^ Neural Induction Medium (NIM) for 5 days to induce neural differentiation to the progenitor cell state. Aggregates containing neural progenitor cells were then seeded onto scaffolds and cultured in NIM for 12 days to induce differentiation to the terminally differentiated cell state.

Bright field images were taken daily of neural aggregates seeded on each set of scaffolds using the IncuCyte^TM^ live imaging platform. The associated software was used to measure the cell body cluster area for each of the 12 day culture period. Cell viability of the neural aggregates seeded on these scaffolds was analyzed after 12 days using a LIVE/DEAD®; Viability/Cytotoxicity Kit (Invitrogen) [24, 25]. Terminal neuronal differentiation of hiPSCs was assessed by performing immunocytochemistry targeting the neuronal protein, Tuj1 [14, 25].

### Model development

**Fig. 3:**
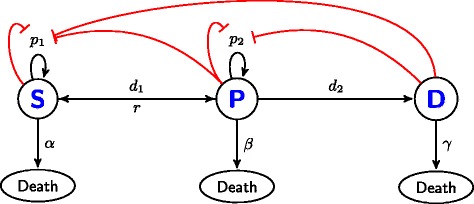
Schematic diagram of the three cell states with cellular feedback. Black arrows indicate transitions between states. Red arrows indicate negative feedback

Figure 3 shows our model system and the interactions between cellular populations. For the derivation of the model, the aggregate cell population was divided into three subpopulations: stem, progenitor and differentiated cells, denoted respectively by *S*, *P*, and *D*, with the total number of cells denoted by *T* where *T* = *S* + *P* + *D*. The feasible region for these variables is *S,P,D*∈[0,*∞*). The rates of the cellular processes are positive (though we allow the possibility *r*=0) and are given as follows:

- *α*: Death rate of stem cells
- *β*: Death rate of progenitor cells
- *γ*: Death rate of differentiated cells
- *p*_1_: Proliferation rate of stem cells
- *p*_2_: Proliferation rate of progenitor cells
- *d*_1_: Differentiation rate of stem cells to progenitor cells
- *d*_2_: Differentiation rate of progenitor cells to differentiated cells
- *r*: Reversion rate of progenitor cells to stem cells.

The units for the above rates are proportion per minute.

All three subpopulations undergo death, but only the stem and progenitor populations proliferate as differentiation was considered terminal. Progenitor cells can revert into stem cells.

The model also includes the oxygen and waste concentrations experienced by the aggregate, denoted by *O* and *W*, respectively, and diffusion from the air-medium interface. *O* and *W* are represented by percentages ranging from 0 to 100. The concentrations of *O* and *W* in the air surrounding the culture are denoted *O*_*air*_ and *W*_*air*_, with the same feasible regions as *O* and *W*. All three cell populations consume oxygen and produce waste. The resulting model is

1 $$  \begin{aligned} \frac{dS}{dt}=&-\alpha S - d_{1}S + \frac{2p_{1}S}{1+S+P+D} +rP\\ \frac{dP}{dt}=&-\beta P -d_{2}P-rP+\frac{2p_{2}P}{1+P+D}+d_{1}S\\ \frac{dD}{dt}=&-\gamma D+d_{2}P\\ \frac{dO}{dt}=&-u_{1}S-u_{2}P-u_{3}D+O_{flux}(S,P,D,O)\\ \frac{dW}{dt}=& w_{1}S+w_{2}P+w_{3}D+W_{flux}(S,P,D,W), \end{aligned}  $$

where the dependence of *S,P*, and *D* on *O* and *W* are through the rate parameters, *α*,*β*,*γ*,*d*_1_,*d*_2_,*p*_1_,*p*_2_, and *r*, and where *u*_*i*_ are the oxygen consumption rates, *w*_*i*_ are the waste production rates, and *O*_*flux*_ and *W*_*flux*_ are the rates of diffusion of *O* and *W* between the neural aggregate and air above the medium.

The units are minutes for time, centimeters for distance, and percentage for gas concentration within the medium.

#### General structure of the model

The “compartments” of this compartmental model consist of the populations of stem, progenitor and differentiated cells along with the concentrations of oxygen and waste. This choice of model was based on a number of considerations. First, these cell states can be distinguished in the lab and cells can be held at each state. Second, each state has unique properties, some of which have been determined experimentally. Finally, the cellular scale is coarse enough that there is useful data for modeling from experimental work and from the literature, but fine enough that the results of the model can be interpreted and transferred to the lab protocol. Each of the cell populations undergoes the appropriate cellular processes for its state. The stem cells can proliferate, differentiate, and die. The progenitor cells undergo four processes: proliferation via division, differentiation to terminally differentiated cells, reversion to stem cells, and cell death. Differentiated cells can only die. Proliferation of earlier states is inhibited by the presence of cells in the same and later states.

Using ordinary differential equations (ODEs) to model cell populations means that they are continuous rather than integer-valued variables, which can cause unrealistic results when cell numbers fall to low values. Here the cell populations number in the hundreds to thousands of cells, limiting any behavioral artifacts that may arise from using ODEs, and in particular, stochastic effects.

Local oxygen and waste concentrations also were incorporated. Oxygen levels influence stem cell proliferation and differentiation [8, 20, 21, 26–28]. Additionally, O_2_ and CO_2_ influence neural stem cell differentiation and these levels can be changed experimentally [8, 20, 21, 26–28]. Thus, including these variables can help determine how to optimize current experimental protocols. The model uses CO_2_ (a cellular waste product that can be measured experimentally) as a proxy for waste.

#### Scaffold modeling

The model incorporates the scaffold properties through a cell-scaffold contact rate, (*C*), which we take to range from 1 to 10, where 1 represents a 90% porous scaffold and 10 represents a solid scaffold, i.e., 0% porosity. This contact rate, *C*, increases with decreasing porosity, but does not go below 1 because we consider lower values to represent the scenario where the cells would not adhere to the substrate. The neural aggregate is roughly spherical, so 100% contact does not mean that all the cells are in contact with the scaffold, but rather that the maximum possible proportion of cells are making contact with the scaffold. This maximum possible contact is estimated heuristically to be about 10%. The relationship between porosity and *C* is

$$\begin{array}{*{20}l} C= (100\%-\text{Porosity})\cdot(0.1), \end{array} $$

where the porosity has units of percent and the factor 0.1 represents the maximum possible contact.

Experimentally, scaffold porosity was decreased by increasing fiber diameter rather than by increasing the density of fibers with the same diameter. Altering porosity in this manner is not optimal as it also changes pore size. It was used in this study because of experimental limitations. We do not explicitly account for changes in fiber diameter and pore size in the model. However, the data used to fit some of the effects of scaffold porosity come from scaffolds with different fiber diameters, so the effects are implicitly included in the model. Another factor related to scaffolds is the topography. The loop mesh scaffolds are 2D scaffolds formed by randomly aligned layers of loop fibers. We did not include the effects of topography because it has previously been optimized experimentally [14] and it would add a significant level of complexity to the model.

The effect of scaffold porosity on differentiation and proliferation is not linear. If the scaffold is too porous, the aggregates cannot adhere, and they fall through the scaffolds gaps. Additionally, a too-porous scaffold does not act on the cells strongly enough to signal for proliferation and differentiation. Conversely, a non-porous scaffold inhibits proliferation because contact inhibition comes into play. A non-porous scaffold does not affect differentiation to the same degree. The effect of contact on differentiation plateaus at a certain rate of contact. These effects are modeled as functional terms in the proliferation and differentiation rates of the stem and progenitor cell variables.

#### Determination of functional effects

Many parameters in Eq. (1) depend on *O*, *W*, and *C* in non-trivial ways. These functional effects multiply the baseline experimental rates for each of the processes. They are included multiplicatively because they are all independent effects. Data were taken from experiments that were testing alteration of only one condition at a time. The qualitative and quantitative data are taken from literature using similar cells and under similar culture conditions in order to minimize differences arising from factors that were not of interest.

**Fig. 4:**
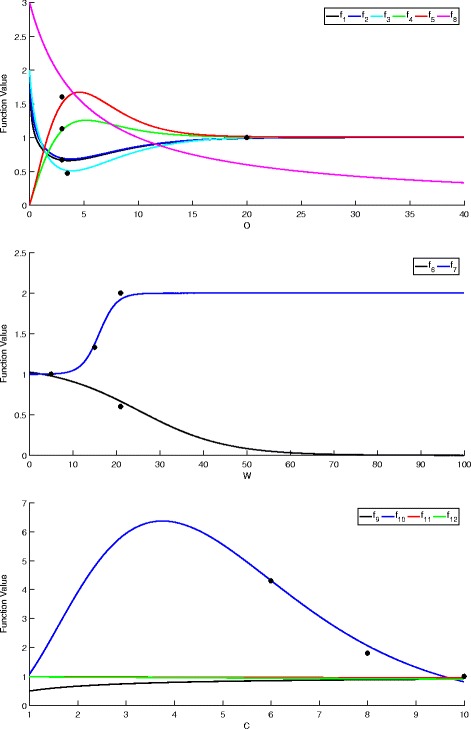
Functional Effects of *O* (top), *W* (middle), and *C* (bottom). When applicable, black markers indicate experimental data used for fitting

Each effect was first determined qualitatively, then fit to quantitative data. The functions were fitted manually because limited data were available. All of the functions for the effects take values greater than 0, where values above 1 increase the rate and values below 1 decrease the rate from the baseline value. Once the functional effects are determined, it is useful for later analysis to find the maximum and minimum values for each. Table 1 gives the individual functional effects, as well as the ranges for each over the appropriate domains of *O*, *W* and *C*. Figure 4 shows the individual effects and Table 2 shows how these functions are built into the parameters.

**Table 1:** Components of functional effects on parameters and extremal values

	Function	Minimum point	Maximum point	Function range
*f*_1_	$\frac {1}{1+10O}-0.3Oe^{-0.3O}+1$	(3.57,0.66)	(0,2)	[0.66,2]
*f*_2_	$\frac {1}{1+5O}-0.3Oe^{-0.3O}+1$	(3.77,0.69)	(0,2)	[0.69,2]
*f*_3_	$\frac {1}{1+2O}-0.5Oe^{-0.3O}+1$	(3.89,0.51)	(0,2)	[0.51,2]
*f*_4_	(0.4*O*−1)(0.4*O*+1)*e* ^−0.5*O*^+1.01	(0,0.01)	(5.2,1.26)	[0.01,1.257]
*f*_5_	(0.6*O*−1)(0.6*O*+1)*e* ^−0.5*O*^+1.01	(0,0.01)	(4.6,1.67)	[0.01,1.67]
*f*_6_	$\frac {10}{9}\left (1-\frac {1}{1+e^{-0.1(W-25)}}\right)$	(100,0)	(0,1.03)	[0,1.03]
*f*_7_	$1+\frac {1}{1+e^{-0.5(W-16)}}$	(0,1)	(100,2)	[1,2]
*f*_8_	$\frac {15}{O+5}$	(100,0.14)	(0,3)	[0.14,3]
*f*_9_	$\frac {C}{C+1}$	(1,0.5)	(100,0.99)	[0.5,0.99]
*f*_10_	4*C* ^3^ *e* ^−0.5−0.8*C*^	(100,0)	(3.75,6.37)	[0,6.37]
*f*_11_	$\frac {200}{200+C}$	(10,0.95)	(1,0.99)	[0.95,0.99]
*f*_12_	$\frac {100}{100+C}$	(10,0.91)	(1,0.99)	[0.91,0.99]
*f*_13_	$\frac {1}{1+e^{-10(O-0.25)}}$	(0,0.076)	(100,1.00)	[0.076,1.00]

**Table 2:** Experimental and compound parameter values

Par	Experimental value range (×10^−5^)	Ref	Functional effect range	Compound value range
$\alpha =\bar {\alpha }f_{1}f_{7}$	[0.1,2.6]^∗^	[14]	[0.66,4]	[0.00000066,0.00010]
$\beta =\bar {\beta }f_{2}f_{7}$	[1.6,2.6]	[14]	[0.685,4]	[0.000011,0.000104]
$\gamma =\bar {\gamma }f_{3}f_{7}$	[1.6,2.6]	[14]	[0.51,4]	[0.00000816,0.000104]
$p_{1}=\bar {p_{1}}f_{5}f_{6}f_{10}$	[69,120]	[7, 29, 37]	[0,11.81]	[0,0.014]
$p_{2}=\bar {p_{2}}f_{5}f_{6}f_{10}$	[45,160]	[7, 29, 37]	[0,11.81]	[0,0.019]
$d_{1}=\bar {d_{1}}f_{4}f_{9}$	[10,17]	[8]	[0.0050,1.24]	[0.00000050,0.00021]
$d_{2}=\bar {d_{2}}f_{4}f_{9}$	[7.3,8.2]	[8]	[0.0050,1.24]	[0.00000036,0.000102]
$r=\bar {r}f_{8}$	[0.1,17]^∗^	[8]	[0.14,3]	[0.00000014,0.00051]

The functional effect is the product of feedbacks for each parameter, e.g. *f*_*α*_=*f*_1_ *f*_7_. The compound value is the experimental value, denoted by a bar above the parameter, multiplied by the functional effect, e.g. $ \bar {\alpha }f_{\alpha }$

^*^No measurements available. Closest related measurements were taken. For *α*, the upper bound for *β* was used. For *r*, the upper bound for *d*_1_ was used. In both cases, it is taken that 0.000001 is the lower bound

The complete parameter coefficients of the model are products of the above functional components and the experimental parameter values.

#### Determination of experimental rates

The experimental rates were determined from multiple sources, including experimental data and data from literature based on similarity to our experimental set-up. Considerations include cell type and culture conditions (O_2_ concentration of 21*%*, CO_2_ concentration of 5%, temperature of 37 °C, etc.).

For each parameter, a combination of the functions in Table 1 multiplies the experimental parameter value to produce the compound parameter value as detailed in Table 2.

In terms of notation, the compound parameter is given by *α*, and the experimental parameter value is referred to as $\bar {\alpha }$, and similarly for the other parameters.

#### Diffusion

Diffusion through the medium from the air to the neural aggregate, and from the neural aggregate to the air, leads to changes in the concentrations of oxygen and waste around the neural aggregate during the experiment. The diffusion of oxygen and waste is affected by the scaffold porosity. Lower porosity scaffolds decrease local diffusion by limiting flow under the aggregate. This effect is modeled by terms multiplying the diffusion terms, but the form of this functional effect could only be estimated, because quantitative data were not available.

**Oxygen and waste equations**

The complete oxygen and waste equations are comprised of consumption and production of oxygen and waste, respectively, by each cell population, and diffusion, occurring in or out depending on the relative concentrations of gases between the local environment, i.e., *O* and *W*, and the external environment, i.e., *O*_*air*_ and *W*_*air*_. The rates for consumption are taken from literature [29] and converted to a percentage as previously described. Each cell population has a specific consumption rate because metabolic requirements vary with differentiation state. Stem cells consume high levels of oxygen [29, 30] as they complete the cell cycle faster than their differentiated counterparts [7, 31, 32]. As cells differentiate, the cell cycle lengthens, and slower cycling times lead to decreased oxygen consumption [7, 31, 32]. Note that the oxygen consumption rates are multiplied by *f*_13_ (see Table 1) to model how cells alter oxygen use during anoxia.

Cells also produce waste products, which affect cellular processes upon accumulation [33, 34]. Thus, cells may slow or arrest proliferation, and even trigger apoptosis in a high waste environment [33, 34]. In this model, we use CO_2_ as a proxy for cellular waste. According to literature, the production of CO_2_ closely matches the consumption of O_2_ (units in mol). Thus, the values for O_2_ consumption were used to determine the CO_2_ production, using a different factor for the mol to % conversion, which was determined in a similar fashion to the oxygen conversion factor. The waste production rates are multiplied by *f*_13_ (see Table 1) to model the way in which cells alter metabolic activity during anoxia.

**Diffusion equations** Typical cell culture medium has a relative density to water of 1.00−1.06 (STEMCELL Technologies, personal communication, June 9, 2016). Thus, we used the density of water (1.00) for our model. The diffusion terms are given by

$$\begin{array}{*{20}l} O_{flux}=&J_{O_{2}}(S,P,D,O)\cdot A_{agg}(S,P,D),\\ W_{flux}=&J_{CO_{2}}(S,P,D,W)\cdot A_{agg}(S,P,D), \end{array} $$

where *J* is the flux and *A*_*agg*_ is the cross-sectional area of the aggregate (see equations in the Appendix). Note that $J_{O_{2}}$ and $J_{CO_{2}}$ are also dependent on the volume of media used and external concentrations of *O*_2_ and *CO*_2_, respectively, which are constant for any given experimental setup. The flux terms for *O* and *W* are multiplied by *f*_10_ and *f*_11_ (see Table 1), respectively, to include the effect of scaffold density on diffusion.

#### Fixed point existence and stability

Analyzing the dynamics of the model gives useful information that can be applied to experimental work. First, we will show that the model predicts convergence to a steady state (fixed point), the final resting state of the growth process.

A fixed point of the model corresponds to a special state in the cell culture that once reached would be maintained, an aggregate with constant size and constant populations of the three cell types. A main goal is to obtain an aggregate with as large a population as possible of a particular cell state. Thus, we wish to maximize the fixed points with respect to a certain cell state. Although ultimately the goal is to produce as large a population, *D*, of differentiated cells as possible, one approach might be to optimize *S* or *P* first, and then introduce a chemical factor to trigger differentiation. In order to achieve an optimal population of one of the cell states, we can alter *O*, *W*, *C*, and differentiation rates, which are accessible by changes to experimental procedures. *O* and *W* can be controlled by altering the O_2_ and C O_2_ levels in the culture chamber. The value of *C* can be altered by changing the porosity of the scaffold on which the cells are seeded. The differentiation rates can be modified by the addition of chemical factors in the medium, either at the start of the experiment, or during the culture period. In the following sections we consider separately optimization of each population and total cell numbers.

### Possible changes to experimental procedure

In addition to scaffold porosity (contact parameter) and seeding protocol (initial populations of each cell type), oxygen serves as a critical factor for experiments affecting stem cell culture. The current experimental procedure uses an oxygen controlled chamber that holds the concentration near ambient levels (∼ 21*%*). Changing the oxygen concentration experienced by the cells affects both differentiation and proliferation [8, 27]. In the case of neural stem cells, the oxygen concentrations in the brain are lower than in the rest of the body, reaching as low as 0.55*%* in some regions of the brain. Other areas of the body maintain up to 9*%* oxygen [20]. Changing the cell culture medium influences cell behavior. Flow chambers can continuously refresh the medium with oxygen and nutrients and remove excess waste products. We determined whether a static culture or a culture with a medium replacement regimen would be optimal by running the model under the conditions where oxygen and waste are taken as variables or held constant.

### Relation of model dimension to experimental conditions

The main goal of this work is to construct and analyze a 5-dimensional model, but analysis of lower dimensional versions of the model allows for a comparison of different experimental procedures. In our experimental work, we attempted to seed with an aggregate consisting only of progenitor cells (so that initially *S*(0)=*D*(0)=0). If reversion of progenitors to the stem cell state is negligible (*r*=0), then the stem cell population remains at zero, and can be excluded from the model. However, we are interested in the effects of reversion and the possibility that some of the initial cells are stem cells. Oxygen and waste can be kept at a constant level experimentally by continuous medium replacement. We therefore analyzed our system under four conditions, each with a different set (and number) of variables:

- The *PD* system refers to the system without stem cells and with *O* and *W* kept constant by medium replacement;
- The *PDOW* system refers to the system without stem cells and with variable *O* and *W* (no medium replacement);
- The *SPD* system refers to the system with stem cells and with *O* and *W* kept constant by medium replacement;
- The *SPDOW* system refers to the system with stem cells and with variable *O* and *W* (no medium replacement).

For the sake of brevity, the systems without stem cells are discussed in the Appendix.

## Results

### Experimental results

Neural aggregates derived from hiPSCs were seeded on two different scaffolds for 12 days. The average results of 3 experiments 12 days after seeding are summarized in Table 3.

**Table 3:** Comparison of data from three experiments for two scaffold porosities 12 days after seeding

Scaffold type	Loop mesh 200	Loop mesh 500
Porosity (%)	40	23
Tuj1 fluorescence (%)	71.5 ± 1	58.4 ± 3
Cell body cluster area (mm^2^)	2.04 ± 0.1	0.87 ± 0.27

**Fig. 5:**
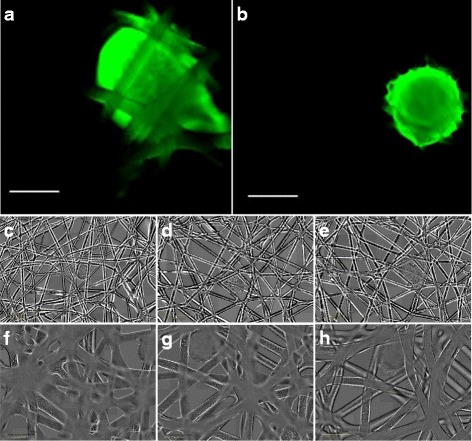
Experimental results. *Top*: Fluorescence images of neuronal marker Tuj1 expressed in neural aggregates 12 days after seeding on loop mesh 200 (**a**) and loop mesh 500 (**b**) scaffolds. Scale bar is 400 *μ*m. *Middle*: Bright field images of neural aggregates on loop mesh 200 scaffolds 0 (**c**), 6 (**d**) and 12 (**e**) days after seeding. *Bottom*: Bright field images of neural aggregates on loop mesh 500 scaffolds 0 (**f**), 6 (**g**) and 12 (**h**) days after seeding

Figure 5 and Table 4 show that both scaffold topographies support cell adhesion and cell migration.

**Table 4:** Cell body cluster area for two neural aggregates seeded on loop mesh 200 and loop mesh 500

	Loop mesh 200		Loop mesh 500	
	Cell body	Number	Cell body	Number
Day	cluster area (mm^2^)	of cells	cluster area (mm^2^)	of cells
0	0.85	5066	0.75	4199
2	0.92	5706	0.78	4454
4	1.11	7561	0.80	4626
6	1.44	11,172	0.88	5337
8	1.67	13,953	0.91	5612
10	1.82	15,874	1.00	6456
12	2.24	21,675	1.1	7459

Number of cells was calculated from cell body cluster area assuming an initial population of 4500 cells (see Appendix)

As shown in Tables 3 and 4, cell body cluster area of neural aggregates cultured on more porous scaffolds was consistently larger than that of neural progenitors seeded on less porous scaffolds.

### Model analysis results

The *SPD* system is comprised of the first three equations of System 1, without the differential equations for *O* and *W*. The corresponding schematic is given in Fig. 3.

Our strategy is as follows. First, we find the fixed points of the system in terms of the parameters, which we do by means of an approximation, and then determine their stability to establish conditions under which a positive fixed point is reached. Since the fixed points depend on the parameters, we can determine how each population is affected by each of the parameters individually, and find the parameter values for which the population is maximized. However, the parameters are not independent in experiments, but depend on *O*, *W*, and *C*, so we next optimize the parameters as functions of *O*, *W*, and *C*, guided by the independently optimized parameter sets. Using the optimal *O* and *W*, we determine the corresponding *O*_*air*_ and *W*_*air*_ in the *SPDOW* model, indicating optimal culture conditions.

#### Existence of fixed points

The fixed points of the 3D system are determined by finding the intersections of the nullclines, given by

$$\begin{array}{*{20}l} 0=&-\alpha S + rP +\frac{2p_{1}S}{1+S+P+D}-d_{1}S\\ 0=&-\beta P - rP +\frac{2p_{2}P}{1+P+D}+d_{1}S-d_{2}P\\ 0=&-\gamma D+d_{2}P. \end{array} $$

(*S,P,D*)=(0,0,0) is one solution to this system. To find a non-zero solution, the three nullclines are solved simultaneously. From the *D* nullcline we have

$$\begin{array}{*{20}l} D=& \frac{d_{2}P}{\gamma}, \end{array} $$

which can be substituted into the equations for the *S* and *P* nullclines. As *D* is only dependent on *P*, finding the intersection of the nullclines for *S* and *P* will be sufficient to find the fixed point. The *P* nullcline, denoted *N*_1_, becomes

$$\begin{array}{*{20}l} P^{2}\left(\frac{\beta+r+d_{2}}{d_{1}}\right)&-SP-S\left(\frac{\gamma}{\gamma+d_{2}}\right)\\ &+P\left(\frac{\gamma(\beta+r+d_{2}-2p_{2})}{d_{1}(\gamma+d_{2})}\right)=0, \end{array} $$

or, equivalently,

$$\begin{array}{*{20}l} &S=P\left(a-\frac{b}{c+P}\right), \end{array} $$

where $a=\frac {\beta +r+d_{2}}{d_{1}}$, $c=\frac {\gamma }{\gamma +d_{2}}$ and $b=\frac {2p_{2}c}{d_{1}}$.

Similarly, the *S* nullcline, denoted *N*_2_, becomes

$$\begin{array}{*{20}l} &S^{2}(\alpha+d_{1})+SP\left(\frac{(\alpha+d_{1})(\gamma+d_{2})}{\gamma}-r\right)\\ -&P^{2}\left(\frac{r(\gamma+d_{2})}{\gamma}\right)+S(\alpha+d_{1}-2p_{1})-rP=0. \end{array} $$

Therefore, the fixed points of the system are given by the intersections of two hyperbolas, and there can be between zero and four in principle. As (*S,P,D*)=(0,0,0) is a known solution, there is at least one intersection, and at most three positive intersections. It can be shown that the nullclines here have a unique positive intersection, the other two being negative in either *S* or *P* (and therefore not meaningful). The *S* value of this positive intersection is an increasing function of the *P* values. Solving the nullclines for a positive fixed point directly becomes too complicated to be useful analytically, so we approximate using the asymptotes of the nullclines for large values of *P*.

The asymptotes of *N*_1_ and *N*_2_, denoted *S*_1*A*_ and *S*_2*A*_ respectively, are determined by ensuring that ${\lim }_{P\to \infty }S-S_{1A}=0$, where *S* is the value of *S* on *N*_1_, and similarly, ${\lim }_{P\to \infty }S-S_{2A}=0$, where *S* is the value of *S* on *N*_2_. The resulting asymptotes are

$$\begin{array}{*{20}l} S_{1A}=&P\left(\frac{\beta+r+d_{2}}{d_{1}}\right)-\frac{2p_{2}\gamma}{d_{1}(d_{2}+\gamma)},\\ S_{2A} =&\frac{Pr}{\alpha+d_{1}}+\frac{2p_{1}r\gamma}{(\alpha+d_{1})[(\alpha+d_{1})(\gamma+d_{2})+r\gamma]}. \end{array} $$

**Fig. 6:**
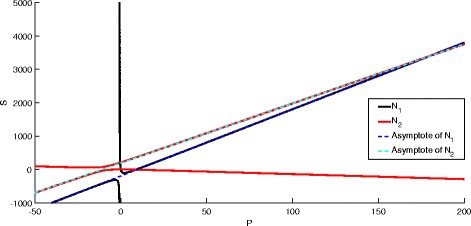
Example of hyperbola and asymptote intersection for *N*_1_ and *N*_2_. Note that the intersection of the asymptotes is near the intersection of the hyperbolas at large *P*

Figure 6 shows an example of the fixed point given by nullcline intersection and the approximate fixed point determined by the asymptote intersection.

The *S*, *P*, *D*, and *T* values at the positive asymptote intersection are

2 $$\begin{array}{*{20}l}{} S^{*}=&\frac{2\gamma r\left[p_{1}(\beta+d_{2}+r)+p_{2}\left(\alpha+d_{1}+\frac{r\gamma}{\gamma+d_{2}}\right)\right]}{[(\alpha+d_{1})(\beta+d_{2})+r\alpha][(\alpha+d_{1})(\gamma+d_{2})+r\gamma]},& \end{array} $$

3 $$\begin{array}{*{20}l} {}P^{*}=& \frac{2\gamma\left[p_{1}rd_{1}+p_{2}(\alpha+d_{1})^{2}+\frac{p_{2}r\gamma(\alpha+d_{1})}{\gamma+d_{2}}\right]}{[(\alpha+d_{1})(\beta+d_{2})+r\alpha][(\alpha+d_{1})(\gamma+d_{2})+r\gamma]}, \end{array} $$

4 $$\begin{array}{*{20}l} {}D^{*}=&\frac{2d_{2}\left[p_{1}rd_{1}+p_{2}(\alpha+d_{1})^{2}+\frac{p_{2}r\gamma(\alpha+d_{1})}{\gamma+d_{2}}\right]}{[(\alpha+d_{1})(\beta+d_{2})+r\alpha][(\alpha+d_{1})(\gamma+d_{2})+r\gamma]}, \end{array} $$

5 $$\begin{array}{*{20}l} {} T^{*}=&\frac{2p_{1}\left[r\gamma(\beta+d_{2})+rd_{1}(\gamma+d_{2})+r^{2}\gamma\right]}{[(\alpha+d_{1})(\beta+d_{2})+r\alpha][(\alpha+d_{1})(\gamma+d_{2})+r\gamma]}\\ +&\frac{2p_{2}\left[(\alpha+d_{1})((\alpha+d_{1})(\gamma+d_{2})+2r\gamma)+\frac{r^{2}\gamma^{2}}{\gamma+d_{2}}\right]}{[(\alpha+d_{1})(\beta+d_{2})+r\alpha][(\alpha+d_{1})(\gamma+d_{2})+r\gamma]}. \end{array} $$

Our fixed points should occur at relatively large values of *P* and *S*, making these approximate values accurate. Since the approximate fixed point values have been expressed explicitly in terms of the parameters, the effects of parameters on the fixed points can be determined, and each population can be optimized with respect to each of the parameters, as was done for the *PD* system. Again, optimization of the individual populations as well as the total population may be of interest, depending upon the goal of the experimentalist.

#### Stability of fixed points

The Jacobian of the *SPD* system is given by

$$\begin{array}{*{20}l} &J(S,P,D)=\\ {}&\left[\begin{array}{ccc} \frac{2p_{1}(1+P+D)}{(1+S+P+D)^{2}}-(\alpha+d_{1}) & r-\frac{2p_{1}S}{(1+S+P+D)^{2}} & \frac{-2p_{1}S}{(1+S+P+D)^{2}}\\ d_{1} & \frac{2p_{2}(1+D)}{(1+P+D)^{2}}-(\beta+d_{2}+r) & \frac{-2p_{2}P}{(1+P+D)^{2}}\\ 0 & d_{2} & -\gamma \end{array}\right]. \end{array} $$

By the Routh-Hurwitz condition, the zero fixed point is stable when 1. 2*p*_1_+2*p*_2_<*α*+*β*+*d*_1_+*d*_2_+*r* and 2. (2*p*_1_−*α*−*d*_1_)(2*p*_2_−*β*−*d*_2_)>(2*p*_1_−*α*)*r*.

and unstable when either of the inequalities is reversed. This implies that the zero fixed point is stable when either 1. 2*p*_1_<*α* and 2*p*_2_<*β*+*d*_2_, or 2. 2*p*_1_<*α* and 2*p*_2_>*β*+*d*_2_ and$r>(2p_{2}-\beta -d_{2})\left (\frac {d_{1}}{\alpha -2p_{1}}+1\right)$, or 3. *α*<2*p*_1_<*α*+*d*_1_ and 2*p*_2_<*β*+*d*_2_ and$r<(\beta +d_{2}-2p_{2})\left (\frac {d_{1}}{2p_{1}-\alpha }-1\right)$,

and not otherwise (neglecting equalities). Thus, the zero fixed point can only be stable if the two proliferation rates are low. It is always stable if the proliferation rate of stem cells is small in relation to their death rate and the proliferation rate of progenitor cells is small in relation to the combined rate of loss of progenitors to death and differentiation. It can still be stable when one of the two proliferation rates is higher, but only under other restrictions. If the proliferation rate of progenitors is higher, then the reversion rate of progenitors to stem cells must also be sufficiently large. On the other hand, if the proliferation rate of stem cells is higher than half their death rate but not as high as half the combined rate of loss of stem cells to death and differentiation, then the zero fixed point is stable only if the reversion rate is sufficiently small. All cases are possible within the parameter ranges given in Table 2. Thus, a stable zero fixed point is possible within our parameter space, though it is unstable at optimal parameter values found below. The stability of the positive equilibrium was determined numerically at the optimal parameter values found below. In all cases it proves to be stable.

#### Optimizing the positive *SPD* fixed point

The effects of parameters on the values of the positive fixed point are analyzed. We identified parameter values for maximizing each subpopulation.

#### Maximizing *S*^∗^

Equation (2) shows immediately that *S* ^∗^ is maximized at maximum allowed values of *p*_1_ and *p*_2_. Differentiating *S* ^∗^ with respect to each of the other parameters shows that the maximal *S* ^∗^ is achieved when *α*, *β*, *d*_1_, and *d*_2_ are minimized, and when *γ* and *r* are maximized.

#### Maximizing *P*^∗^ and *D*^∗^

Equation (3) shows immediately that *P* ^∗^ is maximized when *p*_1_ and *p*_2_ are maximized, and when *β* and *d*_2_ are minimized. Differentiating *P* ^∗^ with respect to each of the other parameters shows that the maximal *P* ^∗^ is achieved when *α* and *r* are minimized (but see below for *r*), when *γ* is maximized, and when $d_{1}=d_{1}^{*}$, an intermediate value in the allowed range for *d*_1_. When the other parameters are at their optimal values, $d_{1}^{*}=0.0000008675$.

The only parameters that differ in *D* ^∗^ from *P* ^∗^ are *d*_2_ and *γ*, so the rest of the optimal parameters remain the same for *D* ^∗^ as for *P* ^∗^. Differentiation with respect to *d*_2_ and *γ* shows that *P* ^∗^ is maximized when *γ* is minimized, and when $d_{2}=d_{2}^{*}$, an intermediate value in the allowed range for *d*_2_. When the other parameters are at their optimal values, $d_{2}^{*}=0.000009495$.

Taking the minimum allowed *r* maximizes *P* ^∗^ and *D* ^∗^. This conclusion depends on the range for *r*, [*r*_min_,*r*_max_]. Considering *P* ^∗^ (and thus *D* ^∗^) as a function of *r*, there is a maximum at a positive *r* value. If we use the previously determined optimal parameter values and consider our ranges for *d*_1_ and *d*_2_, then *r* ^∗^<*r*_min_ where *r*_min_ is taken from Table 1. If we had *r*_min_<*r* ^∗^, then *r* ^∗^ would be the optimal value rather than *r*_min_. This is an important consideration given that the parameter ranges were determined using limited data and may not fully reflect the true values.

#### Maximizing *T*^∗^

From Eq. (5), the optimal *p*_1_ and *p*_2_ for maximizing *T* ^∗^ are their maximum values. The sign of the derivative of *T* ^∗^ with respect to each other parameter determines that, within the given parameter ranges, *T* ^∗^ is maximized when *α*, *β*, *d*_1_, and *d*_2_ are minimized, and when *γ* and *r* are maximized. The conclusions for *d*_1_ and *r* are valid at least when *β*>*α* and *γ*>*α*, which is the case when all other parameters are at their optimal values.

It is notable that the optimal parameter values to maximize the total cell population at equilibrium are the same as those that maximize the stem cell population. However, there is an important difference in the conditions for this result. This parameter set is the optimal set for all positive parameter values when maximizing *S* ^∗^, while the optimal values of *r* and *d*_1_ are dependent on the relative values of *α*, *β* and *γ* when maximizing *T* ^∗^. If the death rate of stem cells, *α*, exceeds the death rates of progenitor and differentiated cells, *β* and *γ* respectively, the optimal values of *r* and *d*_1_ are not the same as for maximizing *S* ^∗^. Therefore, the total cell population and the stem cell population are optimized together, unless the death rate of stem cells surpasses the death rates of the other compartments.

#### Summary of optimization results

For each subpopulation, the optimal parameters are shown in Table 5. The entries with max and min indicate that, for that population, the optimal value for that parameter is the maximum or minimum.

**Table 5:** Summary of optimization results for the *SPD* model

Parameter	Max *S* ^∗^	Max *P* ^∗^	Max *D* ^∗^	Max *T* ^∗^
*α*	Min	Min	Min	Min
*β*	Min	Min	Min	Min
*γ*	Max	Max	Min	Max
*p*_1_	Max	Max	Max	Max
*p*_2_	Max	Max	Max	Max
*d*_1_	Min	$d_{1}^{{*}\mathrm {a}}$	$d_{1}^{{*}\mathrm {a}}$	Min ^a^
*d*_2_	Min	Min	$d_{2}^{{*}\mathrm {a}}$	Min
*r*	Max	Min ^a^	Min	Max ^a^

Note that the parameter sets for max *S* ^∗^ and max *T* ^∗^ are the same

^a^Indicates a condition on the optimal value

#### Optimization with respect to oxygen, waste and contact

The above fixed points are optimized assuming that the parameters can be adjusted independently of each other. This cannot be achieved in experiment because the parameters actually depend on *O*, *W* and *C*. In order to provide useful feedback for experimental protocols, this dependence must be taken into account. Thus, the functional effects of *O*, *W* and *C* on the parameters were optimized by determining the values of *O*, *W* and *C* that most closely result in the optimal parameter set. For example, the value of *p*_2_ should be maximized in all cases, so the optimal value of each functional effect on *p*_2_, i.e., *f*_5_, *f*_6_, and *f*_10_, should be maximized. To maximize *γ*, one has to maximize *f*_3_ and *f*_7_, but there is a trade-off in attempting to maximize *f*_6_ and *f*_7_ with respect to *W* simultaneously. Values of *W* and *C* were estimated from the function graphs to optimally resolve these trade-offs where they occur. For the optimization of each of *P* ^∗^, *D* ^∗^, and *T* ^∗^, the values used were *W*=5 and *C*=3.75. In principle, *W*=0 is optimal, but *W*=5 was taken because this level of C O_2_ also acts as a buffer in the medium [35] and does not greatly affect the functional values compared to *W*=0. Note that *C*=3.75 agrees in a general sense with the experimental results on porosity of the scaffold.

Optimal *O* values cannot easily be read from the function graphs, and had to be determined by numerically finding the value of *O* for which each of the equilibrium values, *S* ^∗^, *P* ^∗^, *D* ^∗^ and *T* ^∗^, takes its maximal value, using the already-determined optimal values of *C* and *W*. The resulting *O* is the optimal oxygen level that should be used in the experimental protocol for maximizing the appropriate equilibrium population.

The relevant functions for optimizing *β* and *p*_2_, namely *f*_2_ and *f*_5_, as well as the relevant function for minimizing *γ*, in order to maximize *D* ^∗^, namely *f*_3_, have optimal values of *O* grouped near a common oxygen value. The relevant function for optimizing *d*_2_, namely *f*_4_, and the relevant function for maximizing *γ*, in order to maximize *P* ^∗^, have zero as the optimal value of *O*. As *d*_2_ takes its optimal value at a different oxygen level than other parameters, it is of interest to consider *d*_2_ separately when determining the optimal *O* as discussed above. Thus, *d*_2_ was taken as a free parameter in the model, with the other parameters remaining functions of *O*. Treating *d*_2_ independently not only increases the equilibrium population of interest, it indicates how critical the value of *d*_2_ is to the dynamics of the system. In terms of experimental procedure, this separation of *d*_2_ from *O* is feasible. The differentiation process represented by *d*_2_ can be modified chemically. Thus, the results from this decoupling of *d*_2_ from *O* could be transferred to the experimental procedure.

For similar reasons, we also considered the effect of allowing *d*_1_ and *r* to be independent, to reflect experimental control of the differentiation rate of stem cells and the reversion rate of progenitors to stem cells.

The fixed point values for the optimized populations are given in Tables 6. The parameters used in calculating these values are the experimental and compound values as given in Table 2. For the dependent parameters, the appropriate optimal *O*, *W*, and *C* for each situation were applied to the experimental parameter values to give the final compound parameter values.

**Table 6:** Summary of *SPDOW* optimized populations

Optimized population	*S* ^∗^	*P* ^∗^	*D* ^∗^	*T* ^∗^	*O* ^∗^	*W* ^∗^	*O*_*air*_	*W*_*air*_
*S* ^∗^, indep	96869.0	165.2	0.6	97034.8	NA	NA	NA	NA
$\bar {S^{*}}$, dep	3383.3	179.2	94.3	3656.8	0.5994	5	1.9622	3.3338
$\hat {S^{*}}$, dep	3385.2	179.2	94.3	3658.7	0.5993	5.0002	1.9622	3.3338
$\bar {S^{*}}$, *d*_1_,*d*_2_ indep	81249.0	252.3	4.7	81506.0	4.1506	5	6.1227	2.5887
$\hat {S^{*}}$, *d*_1_,*d*_2_ indep	81110.0	251.6	4.7	81366.3	4.1499	5.0008	6.1227	2.5887
$\bar {S^{*}}$, *d*_1_,*d*_2_,*r* indep	83213.0	142.3	2.7	83358.0	4.2405	5	6.1682	2.5990
$\hat {S^{*}}$, *d*_1_,*d*_2_,*r* indep	82908.0	141.6	2.6	83052.2	4.2029	5.0020	6.1682	2.5990
*P* ^∗^, indep	1896.3	3428.2	12.0	5336.5	NA	NA	NA	NA
$\bar {P^{*}}$, dep	12.8	147.3	120.4	280.5	0.8544	5	1.2588	4.5059
$\hat {P^{*}}$, dep	19.2	153.3	123.0	295.5	0.8419	5.0152	1.2588	4.5059
$\bar {P^{*}}$, *d*_2_ indep	37.4	2880.5	53.7	2971.6	4.2776	5	4.9933	4.1258
$\hat {P^{*}}$, *d*_2_ indep	37.5	2881.2	53.7	2972.4	4.2776	5.0001	4.9933	4.1258
*D* ^∗^, indep	848.4	883.9	1028.5	2760.8	NA	NA	NA	NA
$\bar {D^{*}}$, dep	1.0	54.1	334.7	389.8	3.9716	5	4.4258	4.4450
$\hat {D^{*}}$, dep	1.3	54.4	336.7	392.4	3.9704	5.0014	4.4258	4.4450

Numerically calculated values of true equilibria are denoted $\hat {S^{*}}$, $\hat {P^{*}}$, and $\hat {D^{*}}$, while approximate values based on asymptotes are denoted $\bar {S^{*}}$, $\bar {P^{*}}$, and $\bar {D^{*}}$

Note that the independent *P* ^∗^ and *D* ^∗^ equilibria are unstable; all other equilibria are stable

#### Including variable oxygen and waste in the model with stem cells

The *SPDOW* model is given in System (1). The addition of the *O* and *W* variables makes a full analysis impossible without using numerical simulations. However, we can use the results obtained in the *SPD* system to draw conclusions about the *SPDOW* system.

**Fig. 7:**
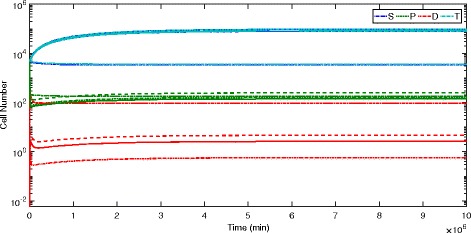
Example of effects of dependence versus independence of parameters on populations. All parameters independent (dot); *d*_1_,*d*_2_,*r* independent (solid); *d*_1_,*d*_2_ independent (dash); all parameters dependent (dash-dot). Parameter set used is for maximizing *S* ^∗^, with *C*=3.75 and *O* ^∗^, *W* ^∗^, *O*_*air*_, and *W*_*air*_ values from Table 6. Experimental parameters (×10^−5^): $ \bar {\alpha }=0.1,\bar {\beta }=1.6, \bar {\gamma }=2.6, \bar {p_{1}}=119, \bar {p_{2}}=160, \bar {d_{1}}=10, \bar {d_{2}}=7.29, \bar {r}=17 $. Independent parameters (×10^−5^): *d*_1_=0.05,*d*_2_=0.03645,*r*=51

In the *SPD* system, we determined the optimal populations and the associated parameters. Since those parameters are functions of oxygen and waste, optimal oxygen and waste concentrations can be calculated from the optimal parameter values. Using the approximate *SPD* fixed point values for *S*, *P*, and *D*, as well as the optimal *O* and *W* values, the *SPDOW* fixed point equations for *O* and *W* can be numerically solved for *O*_*air*_ and *W*_*air*_. It can easily be shown that *O* and *W* must then converge to these fixed point values. Then, with the resulting set of parameter values, the true fixed points, rather than the approximate fixed points determined by the nullcline asymptote intersection, of the *SPDOW* system can be determined numerically. The resulting *SPDOW* fixed points are given in Table 6. The *O*_*air*_ and *W*_*air*_ values required to achieve the optimal *O* and *W* at the equilibrium values of *S*, *P* and *D* are also given in Table 6. The differences between population sizes when various sets of parameters are made independent is further illustrated by the simulations in Fig. 7.

#### Effect of porosity on growth and differentiation

**Fig. 8:**
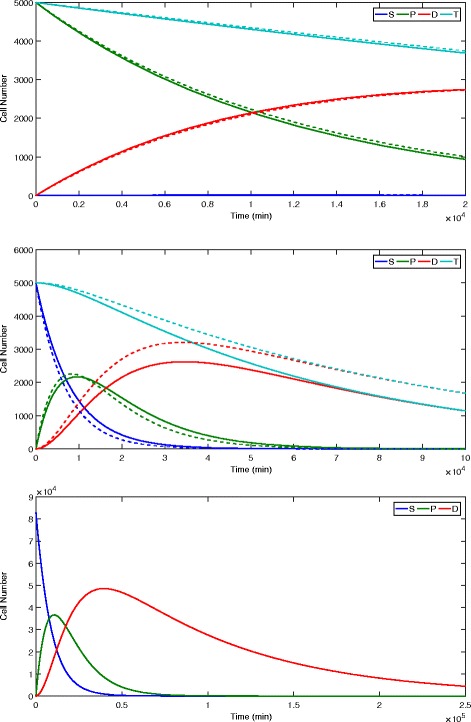
Numerical simulations of population dynamics. *Top*: Population dynamics with initial population of 5000 progenitor cells for *C*=7.7 (solid) and *C*=6 (dash) with *O*=21 and *W*=5. Experimental parameters (×10^−5^): $\bar {\alpha }=0.01,\bar {\beta }=0.16, \bar {\gamma }=0.16,\bar {p_{1}}=119, \bar {p_{2}}=160, \bar {d_{1}}=13.5, \bar {d_{2}}=7.745, \bar {r}=0.1$. *Middle*: Population dynamics with initial population of 5000 stem cells and *C*=10 for *O*=21 (solid) and *O*=5 (dash). Same experimental parameters used as for top simulation. *Bottom*: Population dynamics after switching to parameters for maximizing *D* ^∗^ from the initial point of *S* ^∗^, with independent *d*_1_, *d*_2_, and *r*. See Fig. 7 for parameters used in maximizing *S* ^∗^, and simulation at top for *D* ^∗^ maximizing parameters

Scaffold porosity affects not only differentiation, but also the growth of neural aggregates seeded on the scaffold. As shown in Tables 3 and 4, there is a significant increase in the neural aggregate size when the scaffold porosity is increased from 23 to 40*%*. To compare the experimental results to the model results, we ran simulations of the model with *C*=7.7 (23*%* porosity) and *C*=6 (40*%* porosity). Results are shown in Fig. 8. The dependent parameter set and the experimental parameter set for optimal *D* ^∗^ were used.

#### Oxygen levels

It has also been observed experimentally that culturing neural stem cells in physiologic oxygen conditions (∼ 5*%*) rather than ambient oxygen conditions (∼ 21*%*) increases both cell proliferation and level of differentiation [8]. Because these experiments often used solid substrates for seeding, simulations were run with *C*=10 (0*%* porosity) with dependent parameters and the experimental parameter set for maximal *D* ^∗^ to determine if the model captures these experimental results (Fig. 8). The simulations show that both differentiation and growth increase when oxygen is at the physiologic level of 5*%* versus the ambient oxygen level of 21*%*.

#### Timing of growth and differentiation

The timing of differentiation and growth was also of interest. To determine whether it would be better to grow a large stem cell population, then instigate differentiation, rather than pushing differentiation at seeding, we ran a simulation starting at the *S* ^∗^-optimized equilibrium with parameters set to optimize *D* ^∗^. The results are shown in Fig. 8. *D* rises to a much higher level than in the straightforward *D*-optimizing strategy, but then falls again to settle at an equilibrium value less than this peak level.

## Discussion

In this work, we have developed a mathematical model of human iPSC proliferation and differentiation on scaffolds. In an effort to derive a realistic model, the model development included both qualitative and quantitative data from both laboratory research and previous literature. We included variables and parameters that would not only make interpretation of mathematical and numerical results for experimental procedure possible, but also in order to make optimization of desired outcomes mathematically feasible. By analyzing the model, we are able to explore the effects of changes in parameters that depend on experimental protocol, and thus guide future experiments. In some cases the effects are not intuitively obvious, as there are trade-offs between positive and negative effects of change in even a single parameter.

Computer-aided analysis showed that the model has a unique positive steady state, (*S* ^∗^,*P* ^∗^,*D* ^∗^), to which the system converges over time from any initial set of population values (apart from the inevitable steady state at zero — no cells present). The stem, progenitor, and differentiated cell populations at steady state were optimized with respect to each individual parameter with results given in Table 5. Many of the optimization results agree with intuitive expectations: it makes sense to maximize proliferation and minimize the death rate of stem or progenitor cells in order to maximize their respective equilibrium population sizes. The model analysis also revealed that certain parameters, such as differentiation rates, do not always have such intuitively obvious optimal values. The dependence of the model parameters on oxygen, waste and contact rate imposes additional practical constraints on the accessibility of optimal parameter values in actual experiments. Including these constraints and ways of loosening some constraints by experimental procedure (such as introduction of differentiation factors into the culture medium) allows the mathematical model to give a realistic optimal procedure.

### Maximizing *S*^∗^

One possible objective of an experiment is to maximize the stem cell population, possibly with the idea of later introducing a differentiation factor to drive differentiation. In this case, the model confirms our expectation that the equilibrium stem cell population is maximized at the minimum value of the stem cell death rate (*α*), and at maximum values of the stem cell proliferation rate (*p*_1_) and the reversion rate (*r*). Increasing the reversion not only directly increases stem cells, but also decreases the level of negative feedback to *p*_1_ by decreasing the *P* and *D* populations, and thus also indirectly contributes to increasing *S* ^∗^. Similarly, minimizing *d*_1_ and *d*_2_, the differentiation rates of stem and progenitor cells, decreases the rate of transfer from *S* to *P* and *D*, which decreases the level of inhibitory feedback to *p*_1_ from *P* and *D*, indirectly increasing *S* ^∗^.

The progenitor cell population plays a dual role in relation to the stem cells. It serves as a source of new stem cells via reversion, but inhibits proliferation of stem cells. Thus, it is not clear a priori whether it is better to choose parameters that lead to a higher or lower equilibrium progenitor cell population. Above, we chose *r* and *d*_1_ in a way that detracts from the progenitor cell population but supports the stem cell population directly. However, the model suggests that *p*_2_, the proliferation rate of progenitor cells, and *β*, the death rate of progenitor cells, should be chosen to increase the progenitor cell population. In our model, the reversion overcomes the feedback. However, if the feedback configuration were modified so that the feedback was stronger, then maximizing *p*_2_ and minimizing *β* might not be optimal for maximizing *S* ^∗^. The feedback incorporated into this model is only a possible mechanism, not confirmed in biological reality, so it may be of interest to determine experimentally whether increasing the proliferation rate and decreasing the death rate of *P* increases *S* ^∗^ in reality, which would be consistent with the feedback configuration used in this model, or whether it decreases *S* ^∗^, implying that another feedback mechanism is involved. If the objective is only to maximize the stem cell population, then it would be optimal to maximize *γ*, the death rate of differentiated cells, which only feed back negatively on proliferation of stem and progenitor cells.

In experiments, if we only have control of oxygen level, waste level and contact rate (*O*, *W* and *C*) to alter parameters, then we cannot achieve all the above goals at once. In particular, *γ*, like *α* and *β* is minimized for oxygen levels near 4 or 5%, so if we choose *O*, *W* and *C* to minimize *α* and *β*, then we get a low value of *γ* as well (see Table 2 and Fig. 4). Also, *d*_1_ and *d*_2_ are maximized at oxygen levels near 5% but these are not much lower at 21% oxygen, whereas other parameters vary more with oxygen level, especially the proliferation rates. So, in the numerical optimization with respect to *O* in particular, the disadvantage of having a lower death rate for differentiated cells and slightly higher differentiation rates is outweighed by the advantage of having other parameters at optimal values when oxygen is kept near 5%.

### Maximizing *P*^∗^ and *D*^∗^

If our objective is to maximize the progenitor cell population or the differentiated cell population, it still makes sense to keep a strong pool of stem cells, which are a source of progenitors and thus, indirectly, a source of differentiated cells, and the model confirms that it is best to maximize the proliferation rates *p*_1_ and *p*_2_ and minimize the death rates *α* and *β*. If we were only concerned about maximizing *P* ^∗^, then *γ* should be maximized again, if we could do so in practice, and the reversion rate *r* should be minimized.

An initial intuition suggests that maximizing the rate of differentiation from stem cells to progenitor cells (*d*_1_) would maximize *P* ^∗^ because *d*_1_ directly transfers cells from *S* to *P*. However, because stem cells have a longer life span than progenitor and differentiated cells, as reflected in the values for *α*, *β*, and *γ*, keeping some cells in the stem cell compartment allows for a pool of cells that replenishes the progenitor cell populations. Thus, the optimal value of *d*_1_ is not necessarily its maximum. Here, the optimal *d*_1_ for maximizing *P* ^∗^ and *D* ^∗^ is an intermediate value within the range for *d*_1_ (see Table 1).

As for *d*_1_, the differentiation rate of progenitor cells (*d*_2_) is similarly affected by the range in life spans and we once again get an optimal value of *d*_2_ is not necessarily it’s maximum. The ability of stem cells to proliferate is also a factor in this result. The lack of proliferation in the differentiated cell population means that it is a terminal state, so driving the system to this compartment would drive the populations to extinction eventually. Maintaining *S* ^∗^ and *P* ^∗^ populations allows for more *D* to be formed without forcing the system to the terminal state. Thus, the optimal value of *d*_2_ is not necessarily its minimum. Here, the optimal *d*_2_ for maximizing *D* ^∗^ is an intermediate value within the range for *d*_2_ (see Table 1).

### Maximizing *T*^∗^

The total cell population was optimized along with the stem cell population because optimizing the stem cell population decreases the negative feedback on proliferation and, when *α* is smaller than both *β* and *γ*, minimizes the death rate in the system.

### Controlling differentiation experimentally

If differentiation can be controlled chemically in experiment, it is no longer dependent on oxygen, waste and contact rate like the other parameters. In this case, it is possible to obtain considerably higher steady state populations, whichever subpopulation we wish to optimize (see independent results in Table 6).

### Variable oxygen and waste

The optimal oxygen concentration in the air above the medium is close to or even lower than physiological levels, depending on which population one intends to maximize, while for waste it is around 2.5−5*%*.

When the model parameters are set to match the conditions of the experiments, the population sizes and effects of the change in porosity from 23 to 40*%* are not quantitatively captured, as shown by comparing Table 4 and Fig. 8. The simulated population size is smaller than the sizes seen experimentally. Also, the large difference in population size noted in experiments is not replicated by these simulations. However, the simulations do capture the qualitative effect of increasing population size by decreasing porosity from 23 to 40*%*. It is likely that the lack of quantitative correlation between the experimental data and the model simulations is a result of the parameter estimations used in the model. This discrepancy in quantitative results may not be surprising because the data used to determine both the experimental parameter rates and the functional effects were very limited. Quantitative observations could be captured more effectively with further data collection.

## Conclusions

The simulated dynamics of our model successfully reproduce many experimental observations made about how pluripotent stem cells behave when seeded on biomaterial scaffolds. This model allows investigation of alterations in experimental parameters that would be difficult and costly to explore in the lab. Analysis of the model confirms the existence of a unique and stable positive steady state for plausible parameter ranges. A physiological oxygen level is shown to be optimal whichever subpopulation one wishes to maximize. Discrepancies between quantitative aspects of the model results and experiment could likely be remedied by improved data on rates of cell behavior and their functional dependence on oxygen, waste and contact.

## Appendix

### Flux terms

The explicit flux terms are given by

6 $$ \begin{aligned} O_{flux}=&J_{O_{2}}\cdot \text{Area of aggregate}\\ =&\left[\frac{(3.0\times 10^{-5})\frac{760}{100}(O_{air} -O)(5.1737\times10^{-8})(60)}{d}\right]\pi R^{2}, \end{aligned}  $$

7 $$ \begin{aligned} W_{flux}=&J_{CO_{2}}\cdot \text{Area of aggregate}\\ =& \left[\frac{(2.5\times 10^{-5})\frac{760}{100}(W_{air} -W)(5.1737\times10^{-8})(60)}{d}\right]\pi R^{2} \end{aligned}  $$

where *d*=*H*−*R* is the distance from the top of the medium to the center of the aggregate,

$$H = \frac{V_{\text{medium}}+\frac{4}{3}\pi R^{3}}{9.6} $$

is the height of the medium,

$$R=\left(\frac{S+P+D}{8 \cdot4500 \cdot 1000}\right)^{1/3} $$

is the radius of the aggregate, and where the base of the culture plate has an area of 9.6*cm* ^2^. Note that 3.0×10^−5^ and 2.5×10^−5^ are the diffusion coefficients for *O* and *W*, respectively. The remaining constants in the numerators of the flux terms are unit conversion terms.

### Cell body cluster area to cell number calculation

The number of cells was calculated from cell body cluster area assuming an initial population of 4500 cells. The aggrewell platform used produces aggregates with between 4000 and 5000 cells. With this assumption in place, the calculation of cell numbers from cell body cluster area was determined as follows.

For any time *t*, we have that the approximate number of cells in the aggregate, *N*(*t*), is given by

$$N(t)=CD_{o} \cdot V(t)=\left(\frac{6(4500)}{\pi} \frac{\text{cells}}{mm^{3}}\right)\left(\frac{4}{3}\pi \sqrt{\frac{A(t)}{\pi}}\right) $$

where *A*(*t*) is the experimentally determined cell body cluster area, *V*(*t*) is the volume of the aggregate (derived from *A*(*t*)), and *CD*_*o*_ is the cell density of the aggregate, which is assumed to be constant. We first determine the initial volume of cells using an averaged cell body cluster area of 0.8*mm* ^2^:

$$\begin{array}{*{20}l}{} 0.8mm^{2}=A(0)&=\pi r(0)^{2} \implies r(0)=0.5mm\\ &\qquad\qquad\implies V(0)=\frac{4}{3}\pi (0.5 mm)^{3}, \end{array} $$

where *r*(0) is the radius of the aggregate at *t*=0. Thus, the cell density is given by

$$\begin{array}{*{20}l} CD_{o}=&\frac{4500 \text{\ cells}}{V(0)}=\frac{6(4500) \text{\ cells}}{\pi mm^{3}}. \end{array} $$

Finally, to convert cell body cluster area to number of cells, we have *N*(*t*)=*CD*_*o*_·*V*(*t*).

### The model without stem cells

This version of the model corresponds to the larger model in the case where the initial stem cell population is zero and the reversion rate, *r*, is zero. This reduces it to a two-variable model, for which more analytic tools are available.

The simplified *PD* model is

8 $$ \begin{aligned}  \frac{dP}{dt}=&-\beta P + \frac{2p_{2} P}{1+P+D}-d_{2}P\\ \frac{dD}{dt}=&-\gamma D+ d_{2}P. \end{aligned}  $$

It is more analytically tractable than the *SPD* system, but our analysis strategy is the same.

First, it can be shown that

9 $$\begin{array}{*{20}l}  P\in& \left[0,\frac{2p_{2}}{\beta+d_{2}}\right] \text{and}\ D\in \left[0,\frac{2p_{2}d_{2}}{\gamma(\beta+d_{2})}\right] \end{array} $$

is invariant for the *PD* system and that solutions starting outside this region in the non-negative quadrant fall into it. Also, there are no periodic solutions by Dulac’s Criterion [36].

#### Existence and stability of fixed points

The nullclines of *P* and *D*, the intersection of which determine the fixed points, are given by setting the right hand sides of Eq. (8) to zero. Solving this system gives two fixed points: (0,0), and a unique positive fixed point (*P* ^∗^,*D* ^∗^), which exists if and only if 2*p*_2_>*β*+*d*_2_, where

10 $$\begin{array}{*{20}l} P^{*}=& \frac{\gamma(2p_{2}-\beta-d_{2})}{(\beta+d_{2})(\gamma+d_{2})},  \end{array} $$

11 $$\begin{array}{*{20}l} D^{*}=&\frac{d_{2}(2p_{2}-\beta-d_{2})}{(\beta+d_{2})(\gamma+d_{2})},  \end{array} $$

12 $$\begin{array}{*{20}l} T^{*}=&P^{*}+D^{*}=\frac{2p_{2}}{\beta+d_{2}}-1.  \end{array} $$

The existence condition for the positive fixed point is feasible within the parameter space defined in Table 2. Also, in our parameter space min*p*_2_=0, which means that the situation where the positive fixed point does not exist is also possible.

The Jacobian matrix of the *PD* system is

$$J(P,D)=\left[\begin{array}{cc}-\beta-d_{2}+\frac{2p_{2}(1+D)}{(1+P+D)^{2}} & -\frac{2p_{2}P}{(1+P+D)^{2}} \\ d_{2} & -\gamma \end{array} \right] $$

and it is easy to check that the zero equilibrium is a stable node for 2*p*_2_<*β*+*d*_2_ and is a saddle point, and therefore unstable, for 2*p*_2_>*β*+*d*_2_. It is also easy to show that Trace (*J*)<0 and Det (*J*)>0 for the positive equilibrium (*P* ^∗^,*D* ^∗^) whenever it exists, i.e., when 2*p*_2_>*β*+*d*_2_. In fact, the positive fixed point is globally stable with respect to the invariant region (9) apart from the *D*-axis. This follows from the lack of periodic solutions and the Poincaré-Bendixson Theorem applied to the interior of the invariant region. Thus, when the positive fixed point exists, all solutions converge to it. Otherwise, all solutions go to zero.

It should be noted that although our model precludes solutions reaching the *D*-axis from elsewhere when the positive fixed point exists (i.e., precludes an initially positive *P* from reaching 0), the model is not really appropriate for very small populations, so if *P*=1 or a very small number, then in reality we might expect stochastic effects to allow *P* to jump to 0. This is not a real problem, however, for the ranges of cell numbers we deal with in our experimental setup.

#### Optimizing the positive *PD* fixed point

As for the *SPD* model, we consider each subpopulation, or the total population, at the fixed point as a function of each parameter, and explore the effects of individual parameters on the steady state (sub)populations.

From Eq. (12), the total cell population at equilibrium, *T* ^∗^, is maximized when *p*_2_ is maximized, and when *β* and *d*_2_ are minimized. The parameter *γ* has no effect on *T* ^∗^.

The progenitor cell population at equilibrium, *P* ^∗^, given by Eq. (10) is increasing in *p*_2_ and *γ*, and decreasing in *β* and *d*_2_, as can be determined by considering the sign of the derivative with respect to each parameter. Thus, the progenitor cell population at equilibrium, *P* ^∗^, is maximized when *p*_2_ and *γ* are maximized, and when *β* and *d*_2_ are minimized.

Equation (11) shows that the differentiated cell population at equilibrium, *D* ^∗^, is increasing in *p*_2_ and decreasing in *β* and *γ*, but is neither a strictly increasing or decreasing function of *d*_2_. Solving $\frac {dD^{*}}{dd_{2}}=0$ for positive values of *d*_2_ gives

$$ d_{2}=\frac{-\beta\gamma + \sqrt{\beta^{2}\gamma^{2}+\beta\gamma(2p_{2}+\gamma)(2p_{2}-\beta)}}{2p_{2}+\gamma} $$

at which *D* ^∗^ has a maximum. We denote this value as $d_{2}^{*}$, which, for most values of *p*_2_, *γ*, and *β*, lies in the allowable range for *d*_2_.

The optimal values of the parameters for the PD model are given in Table 7. The optimized fixed points, that is, the values calculated by including the optimal parameters within our given parameter ranges in Eqs. (10) and (11), are given in Table 8. The populations under optimization of *T* ^∗^ are given both for the minimum and maximum values of *γ*, since *T* ^∗^ does not depend on *γ*, though *P* ^∗^ and *D* ^∗^ do.

**Table 7:** Summary of optimal parameter values for maximizing cell populations

Parameter	Max *P* ^∗^	Max *D* ^∗^	Max *T* ^∗^
*β*	Min	Min	Min
*γ*	Max	Min	No effect
*p*_2_	Max	Max	Max
*d*_2_	Min	$d_{2}^{*}$	Min

**Table 8:** Optimized fixed points with *O*-independent parameters

Optimized value	*P* ^∗^	*D* ^∗^	*T* ^∗^	*D* ^∗^/*T* ^∗^
*P* ^∗^	3325.2	11.7	3336.9	0.0035
*D* ^∗^	857.5	993.3	1850.8	0.5367
*T* ^∗^, min *γ*	3194.2	142.7	3336.9	0.0428
*T* ^∗^, max *γ*	3325.2	11.7	3336.9	0.0035

The above fixed points are not experimentally realizable since the parameters are not independent, so we applied the same optimization process as for the *SPD* system taking into account the dependence of the parameters on *O*, *W*, and *C* (Table 9).

**Table 9:** Optimized fixed points for the *PD* system with dependent parameters

Optimized value	*P* ^∗^	*D* ^∗^	*T* ^∗^	*D* ^∗^/*T* ^∗^	*O* ^∗^	*O*_*air*_	*W*_*air*_
*P* ^∗^, coupled	141.6	123.0	264.6	0.4648	0.8976	1.2813	4.5312
*P* ^∗^,*d*_2_ uncoupled	2856.1	53.2	2909.3	0.0183	4.2896	4.9931	4.1407
*D* ^∗^, coupled	53.3	330.7	384.1	0.8611	4.0059	4.4573	4.4485
*D* ^∗^,*d*_2_ uncoupled	908.2	713.2	1621.4	0.4399	4.3585	4.9996	4.2168
*T* ^∗^, coupled, *γ*_min_	60.4	345.0	405.3	0.8511	3.7393	4.1973	4.4404
*T* ^∗^, coupled, *γ*_max_	89.7	315.6	405.3	0.7786	3.7393	4.1932	4.4454
*T* ^∗^,*d*_2_ uncoupled, *γ*_min_	2823.8	85.5	2909.4	0.0294	4.2891	4.9973	4.1394
*T* ^∗^,*d*_2_ uncoupled, *γ*_max_	2856.1	53.2	2909.4	0.0183	3.7393	4.9926	4.1407

Note that *W* ^∗^=5 in all cases. “Coupled” indicates dependence of the parameters on *O*, *W* and *C*. “Uncoupled” indicates that the independent optimal parameter value was used. For the optimal *T* ^∗^ populations, the populations are given for the minimum and maximum values of *γ*, *γ*_min_ and *γ*_max_, because *T* ^∗^ does not depend on *γ*, though *P* ^∗^ and *D* ^∗^ do

**Including variable oxygen and waste in the model without stem cells**

The *PDOW* system is given by

13 $$ \begin{aligned}  \frac{dP}{dT}=&-\beta P +\frac{2p_{2}P}{1+P+D}-d_{2}P\\ \frac{dD}{dt}=&-\gamma D+d_{2}P \\ \frac{dO}{dt}=&-u_{2}P-u_{3}D+O_{flux}(P,D,O)\\ \frac{dW}{dt}=& w_{2}P+w_{3}D+W_{flux}(P,D,W). \end{aligned}  $$

In the *PD* system, we determined the optimal populations and the associated parameters, as well as optimal oxygen and waste concentrations. Using the *PD* fixed points and including the optimal *O* and *W* values as the *O* and *W* components of the *PDOW* fixed point, the fixed point equations for *O* and *W* can be solved for *O*_*air*_ and *W*_*air*_.

The fixed points for *P* and *D* in the *PDOW* system are still those given in Eqs. (10) and (11), and the corresponding value of *T* ^∗^ is still the *γ*-independent value given in Eq. (12). Numerical values of all the variables at fixed points corresponding to the various optimization schemes, are given in Table 9. It can also be shown (from eigenvalues of the Jacobian matrix) that these fixed points are all locally asymptotically stable. The *O*_*air*_ and *W*_*air*_ values required to achieve the optimal *O* and *W* at the equilibrium values of *P* and *D* are also given in Table 9.

### Discussion of the model without stem cells

**Fixed point existence and stability** The model without stem cells always has a fixed point at zero, as expected in a population model. When 2*p*_2_<*β*+*d*_2_, the zero fixed point is stable and there are no other equilibria in the feasible region, so the system will eventually decay to 0 from any initial point. Biologically, 2*p*_2_≤*β*+*d*_2_ corresponds to the situation where the production of progenitor cells by proliferation is overcome by the loss of progenitor cells by death and differentiation. Differentiated cells are a terminal state and inhibit the proliferation of progenitor cells, so the system is driven to extinction.

At 2*p*_2_=*β*+*d*_2_, there is a bifurcation above which the zero fixed point becomes unstable and a stable positive fixed point, (*P* ^∗^,*D* ^∗^), is formed. Thus, for 2*p*_2_>*β*+*d*_2_, both progenitor and differentiated cells persist. In this case, the proliferation rate overcomes the loss of progenitor cells by death and differentiation, allowing for the maintenance of a pool of progenitor cells. This pool of progenitor cells also maintains a population of differentiated cells as the progenitor cells transition to the differentiated state.

**Fixed point dependence on parameters** The progenitor cell population at equilibrium, *P* ^∗^, is maximized when *β* and *d*_2_, the death rate and differentiation rate of progenitors, are minimized, and when *p*_2_ and *γ*, the proliferation rate of progenitors and the death rate of differentiated cells, are maximized. The total cell population, *T* ^∗^, is maximized under the same conditions except that in this case the death rate of differentiated cells has no effect. The differentiated cell population at equilibrium, *D* ^∗^, is maximized when *β* and *γ*, the two death rates, are minimized, when *p*_2_, proliferation of progenitors, is maximized, and when the differentiation rate is at an optimal intermediate value, $d_{2}=d_{2}^{*}$. The proportion of *D* ^∗^ is maximized when *γ* is minimized and *d*_2_ is maximized, while *p*_2_ and *β* do not affect the proportion.

Progenitor cells are the source of new cells in the 2-dimensional system, so the optimal value of *p*_2_ and *β* for optimization of all populations are the maximum and minimum values, respectively. The optimal values of *p*_2_ and *β* have less complicated implications than the other parameters as progenitor cells only feed back on their own proliferation. To maximize any cell population, maximizing the production and minimizing the removal of the proliferative progenitor cell population must be optimal as it supplies both progenitor and differentiated cells. Decreasing the differentiation rate of progenitor cells will increase both progenitor and total cell populations. This effect is a result of two factors. First, the differentiated cell state is terminal. Therefore, prolonging the length of time the cells stay in the proliferative progenitor state (by decreasing *d*_2_) increases the rate of production of new cells. Secondly, the differentiated cell population negatively feeds back onto the proliferation of the progenitor cells. Thus, as *d*_2_ increases and progenitor cells are driven to the differentiated cell population, the inhibition of progenitor cell proliferation increases and will lead to a smaller population of progenitor cells and total cell population.

A different *d*_2_ value maximizes the differentiated cell population: *d*_2_=0.0000094522. This numerical value depends on the values of the other parameters. Thus, if the experimental ranges for parameters were altered so that the optimal values of the parameters were also changed, this optimal *d*_2_ value would be different. For our set of experimental parameters, the optimal *d*_2_ values is low within the range for the compound *d*_2_ parameter. This result may not be immediately obvious because an an initial intuition might be to maximize the rate of creation of differentiated cells in order to maximize *D* ^∗^. However, the differentiated cell state is a terminal state, so the optimal *d*_2_ occurs where there is a balance between creation of differentiated cells and the maintenance of the progenitor cell pool that leads to differentiated cells. The differentiated cell population is maximized by keeping a sizable progenitor pool to produce more differentiated cells. This progenitor cell population is maintained by not driving the population too quickly to the differentiated state as well as keeping the progenitor cell population large enough to overcome the proliferative inhibition from the differentiated cell population. Thus, the size of *D* ^∗^ is maximized at this intermediate value of *d*_2_.

The parameter *γ* is also somewhat counterintuitive. Increasing the death rate of differentiated cells increases the progenitor cell population because fewer differentiated cells mean less inhibition of proliferation of the progenitor cells. Minimizing *γ* also maximizes *D* ^∗^. This is not as obvious as it seems because of the feedback. Although a lower death rate increases the differentiated cell population, it also increases the negative feedback on the proliferation of progenitor cells, which are the source of differentiated cells. Thus, minimizing *γ* is not a priori the correct method for maximizing *D* ^∗^. However, with the current feedback mechanism, the decreased loss of differentiated cells outweighs the decrease in input from the progenitor cell population. Therefore, at least with the current feedback mechanism, minimizing *γ* maximizes *D* ^∗^.

The results of optimization for *p*_2_ and *β* are obvious biologically and are not complicated by other factors: to increase a population, increase proliferation and decrease death. The model is consistent with this observation. The more counterintuitive results for *d*_2_ and *γ* provide more insight into the dynamics of the system.

**Fixed point optimization**

The optimization of the parameters was first carried out assuming each parameter could be altered independently, in order to understand how the populations were affected by each parameter. However, the parameters are actually determined by oxygen, waste and scaffold porosity, and as such cannot be considered independent. Thus, to provide useful information to experimentalists, the populations were optimized with respect to *O,W* and *C* by determining which values resulted in the closest parameter set to the optimal set. The resulting *O* values indicated that a physiological O_2_ level, or lower, is the best for maximizing the populations because most parameters were optimized at these *O* values, while *d*_2_ was the only clear exception. As *d*_2_ was an outlier, the above optimization was also carried out when *d*_2_ was optimized as an independent parameter (corresponding to altering the differentiation rate of progenitors by appropriate chemical factors in experiments). The resulting optimal oxygen level was higher when *d*_2_ was uncoupled, although still within the physiological range. It is also of note that the population levels with *d*_2_ decoupled are significantly higher than the fully coupled counterparts. The optimal *W* was determined to be lower than the typical C O_2_ levels used experimentally. The contact rate *C* determined to be optimal for all populations was 3.75, which agrees in a general sense with the fit to the experimental data.

The population sizes of the fixed points are much smaller than the populations in the experimental data. This may be a consequence of the limited data used for both rate determination and fitting, and consequent inaccuracy in those values. Another possible reason for this discrepancy is that stem cells were actually present in the experiments, despite the intention of seeding only progenitors. The presence of stem cells in the seeded population tends to increase the total cell numbers over time.

## Abbreviations

hiPSC: Human induced pluripotent stem cell
iPSC: Induced pluripotent stem cell
NIM: Neural induction medium
ODE: Ordinary differential equation
PCL: Poly-(*𝜖*-caprolactone)

## References

[1] Langer R, Vacanti J (1993). Tissue engineering. Science.

[2] Willerth SM, Sakiyama-Elbert SE (2007). Approaches to neural tissue engineering using scaffolds for drug delivery. Adv Drug Deliv Rev.

[3] Narsinh KH, Plews J, Wu JC (2011). Comparison of human induced pluripotent and embryonic stem cells: Fraternal or identical twins?. Mol Ther.

[4] Takahashi K, Yamanaka S (2006). Induction of pluripotent stem cells from mouse embryonic and adult fibroblast cultures by defined factors. Cell.

[5] Keung AJ, de JuanPardo EM, Schaffer DV, Kumar S (2011). Rho gtpases mediate the mechanosensitive lineage commitment of neural stem cells. Stem Cells.

[6] Keung AJ, Dong M, Schaffer DV, Kumar S (2013). Pan-neuronal maturation but not neuronal subtype differentiation of adult neural stem cells is mechanosensitive. Sci Rep.

[7] Roccio M, Schmitter D, Knobloch M, Okawa Y, Sage D, Lutolf MP (2013). Predicting stem cell fate changes by differential cell cycle progression patterns. Development (Cambridge, England).

[8] Studer L, Csete M, Lee SH, Kabbani N, Walikonis J, Wold B, McKay R (2000). Enhanced proliferation, survival, and dopaminergic differentiation of cns precursors in lowered oxygen. J Neurosci.

[9] Lane SW, Williams DA, Watt FM (2014). Modulating the stem cell niche for tissue regeneration. Nat Biotechnol.

[10] Lo WC, Chou CS, Gokoffski KK, Wan FY-M, Lander AD, Calof AL, Nie Q (2009). Feedback regulation in multistage cell lineages. Math Biosci Eng.

[11] Herberg M, Roeder I (2015). Computational modelling of embryonic stem-cell fate control. Development.

[12] Jin K, Mao XO, Batteur S, Sun Y, Greenberg DA (2003). Induction of neuronal markers in bone marrow cells: differential effects of growth factors and patterns of intracellular expression. Exp Neurol.

[13] Willerth SM (2011). Neural tissue engineering using embryonic and induced pluripotent stem cells. Stem Cell Research & Therapy.

[14] Mohtaram NK, Ko J, King C, Sun L, Muller N, Jun MB-G, Willerth SM (2015). Electrospun biomaterial scaffolds with varied topographies for neuronal differentiation of human-induced pluripotent stem cells. J Biomed Mater Res A.

[15] Takahashi K, Yamanaka S (2006). Induction of pluripotent stem cells from mouse embryonic and adult fibroblast cultures by defined factors. Cell.

[16] Pagliara S, Franze K, McClain CR, Wylde GW, Fisher CL, Franklin RJM, Kabla AJ, Keyser UF, Chalut KJ (2014). Auxetic nuclei in embryonic stem cells exiting pluripotency. Nat Mater.

[17] Robinson M, Yau S-y, Sun L, Gabers N, Bibault E, Christie BR, Willerth SM (2015). Optimizing differentiation protocols for producing dopaminergic neurons from human induced pluripotent stem cells for tissue engineering applications. Biomarker Insights.

[18] Ko J, Mohtaram NK, Ahmed F, Montgomery A, Carlson M, Lee PC, Willerth SM, Jun MB (2014). Fabrication of poly (*ε*-caprolactone) microfiber scaffolds with varying topography and mechanical properties for stem cell-based tissue engineering applications. J Biomater Sci Polym Ed.

[19] Engler AJ, Sen S, Sweeney HL, Discher DE (2006). Matrix elasticity directs stem cell lineage specification. Cell.

[20] Mohyeldin A, Garón-Muvdi T, Quiõnes-Hinojosa A (2010). Oxygen in stem cell biology: A critical component of the stem cell niche. Cell Stem Cell.

[21] Vieira HLA, Alves PM, Vercelli A (2011). Modulation of neuronal stem cell differentiation by hypoxia and reactive oxygen species. Prog Neurobiol.

[22] Zhu J, Aja S, Kim E, Park MJ, Ramamurthy S, Jia J, Hu X, Geng P, Ronnett GV (2012). Physiological oxygen level is critical for modeling neuronal metabolism in vitro. J Neurosci Res.

[23] Braam SR, Zeinstra L, Litjens S, Ward-van Oostwaard D, van den Brink S, van Laake L, Lebrin F, Kats P, Hochstenbach R, Passier R (2008). Recombinant vitronectin is a functionally defined substrate that supports human embryonic stem cell self-renewal via *α**v**β*5 integrin. Stem Cells.

[24] Montgomery A, Agbay A, Edgar J, Gabers N, Gomez J, Mohtaram N, King C, Mitchell A, Rajwani A, Rattray D, Robinson M, Shapka A, Sun L, Wong A, Willerth S (2014). Combining protein-based biomaterials with stem cells for spinal cord injury repair. OA Stem Cells.

[25] Montgomery A, Wong A, Gabers N, Willerth SM (2015). Engineering personalized neural tissue by combining induced pluripotent stem cells with fibrin scaffolds. Biomater Sci.

[26] Clarke L, van der Kooy D (2009). Low oxygen enhances primitive and definitive neural stem cell colony formation by inhibiting distinct cell death pathways. Stem Cells (Dayton, Ohio).

[27] Ezashi T, Das P, Roberts RM (2005). Low o2 tensions and the prevention of differentiation of hes cells. Proc Natl Acad Sci U S A.

[28] Gray DR, Chen S, Howarth W, Inlow D, Maiorella BL (1996). Co2 in large-scale and high-density cho cell perfusion culture. Cytotechnology.

[29] Birket MJ, Orr AL, Gerencser AA, Madden DT, Vitelli C, Swistowski A, Brand MD, Zeng X (2011). A reduction in atp demand and mitochondrial activity with neural differentiation of human embryonic stem cells. J Cell Sci.

[30] Cho CH, Park J, Nagrath D, Tilles AW, Berthiaume F, Toner M, Yarmush ML (2007). Oxygen uptake rates and liver-specific functions of hepatocyte and 3t3 fibroblast co-cultures. Biotech Bioeng.

[31] Salomoni P, Calegari F (2010). Cell cycle control of mammalian neural stem cells: putting a speed limit on g1. Trends Cell Biol.

[32] Hardwick LJ, Philpott A (2014). Nervous decision-making: to divide or differentiate. Trends Genet.

[33] Altman BJ, Rathmell JC (2012). Metabolic stress in autophagy and cell death pathways. Cold Spring Harb Perspect Biol.

[34] Vander Heiden MG, Cantley LC, Thompson CB (2009). Understanding the warburg effect: the metabolic requirements of cell proliferation. Science.

[35] Scientific TF. pH and CO2 Levels. https://www.thermofisher.com/ca/en/home/references/gibco-cell-culture-basics/cell-culture-environment/ph-co2-levels.html. Accessed 29 June 2016.

[36] Strogatz SH (2014). Nonlinear Dynamics and Chaos: with Applications to Physics, Biology, Chemistry, and Engineering..

[37] Guarino RD, Dike LE, Haq TA, Rowley JA, Pitner JB, Timmins MR (2004). Method for determining oxygen consumption rates of static cultures from microplate measurements of pericellular dissolved oxygen concentration. Biotechnol Bioeng.

